# Combined treatment for Coats’ disease: retinal laser photocoagulation combined with intravitreal bevacizumab injection was effective in two cases

**DOI:** 10.1186/1471-2415-14-36

**Published:** 2014-03-25

**Authors:** Aya Kodama, Koji Sugioka, Shunji Kusaka, Chota Matsumoto, Yoshikazu Shimomura

**Affiliations:** 1 Department of Ophthalmology, Kinki University Faculty of Medicine, 377-2, Ohnohigashi, Osaka-Sayama City, Osaka 589-8511, Japan; 2 Department of Ophthalmology, Sakai Hospital Kinki University Faculty of Medicine, 2-7-1, Harayamadai, Minami Ward, Sakai City, Osaka 590-0132, Japan

**Keywords:** Coats’ disease, Photocoagulation, Bevacizumab, Vascular endothelial growth factor, Exudative retinal detachment

## Abstract

**Background:**

The exact pathogenetic mechanisms of Coats’ disease remain unknown. In this report, we show two cases of Coats’ disease that achieved a favorable prognosis with the combined treatment of intravitreal bevacizumab (IVB) injection prior to photocoagulation, although both initially resisted photocoagulation therapy.

**Case presentations:**

Case 1 was a 15-year-old boy with initial visual acuity of 0.4 OD. At the temporal retina, aneurysms and abnormal telangiectatic vessels were observed. Hard exudates and an exudative retinal detachment extended to the fovea. He was diagnosed as having Coats’ disease at stage 3A and we performed laser photocoagulation as an initial approach to treat peripheral aneurysms and telangiectatic vessels. After the treatment, the exudative retinal detachment was eased and visual acuity improved to 1.0; however, recurrence occurred after 5 months. The exudative change was resistant against laser photocoagulation therapy and we therefore added IVB as an adjuvant before photocoagulation. Fourteen days after IVB injection phased laser photocoagulation was given to cover the abnormal capillaries, aneurysms and the leakage area spotted in FA. A good prognosis was obtained with decreased exudation and improved visual acuity.

Case 2 was an 11-year-old boy with decreased visual acuity of 0.15 OS at the initial visit. Hard exudates, retinal edema and serous retinal detachment were seen at the macula and peripheral retina. Fluorescein angiography revealed telangiectatic capillaries at the temporal retina. Our diagnosis was Coats’ disease at stage 3A. Extensive photocoagulation was performed as an initial treatment to the lesion. However, the exudative change was severe and resistant against the photocoagulation treatment. Therefore, we added IVB as an adjuvant before photocoagulation. Exudative change in the retina seemed to be eased 7 days after IVB injection, therefore, phased laser phototherapy was added to cover the abnormal capillaries. After the combination therapy, exudative change was remarkably ameliorated and better visual acuity was achieved.

**Conclusion:**

Bevacizumab is considered an effective adjuvant for Coats’ disease with exudative change resistant to retinal photocoagulation therapy.

## Background

Coats’ disease is characterized by abnormal telangiectasia, aneurysms of retinal vessels, ensuing retinal exudation and serous retinal detachment [1,2].

The exact pathogenetic mechanisms of Coats’ disease remain unknown, and there is no natural remission. Prognosis is poor if left untreated. A first choice for treating Coats’ disease is laser photocoagulation or cryotherapy for aneurismal and telangiectatic vessels. However, laser photocoagulation is not very effective for cases with severe exudative changes and, the prognosis depends on the presence or absence of exudative retinal detachment. Cases that have severe exudative changes in the retina are often resistant to laser therapy and progress to an advanced level of subretinal exudation or fibrosis, which necessitates cryotherapy with surgical drainage of sub-retinal fluid (SRF) or pars plana vitrectomy [3].

Recently, involvement of vascular endothelial growth factor (VEGF) in Coats’ disease has been discussed. It was reported that, in Coats’ disease, the VEGF concentration was elevated in the fluid of the anterior chamber and vitreous [4,5]. Also, some reports suggested anti-VEGF agents were effective for treating Coats’ disease [6-11].

This article describes two cases of Coats’ disease in which bevacizumab intravitreal (IVB) injection combined with retinal photocoagulation treatment was effective. Although both cases were resistant to coagulation therapy alone, a favorable prognosis was obtained after the addition of IVB prior to laser phototherapy.

## Case presentation

**Figure 1:**
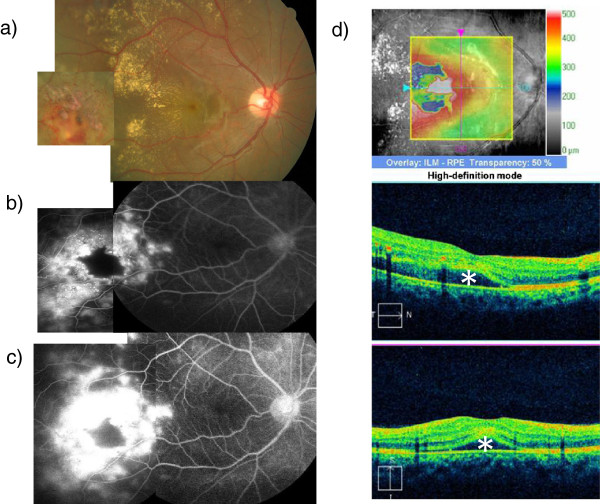
**Case 1 at the initial visit. a)** Fundus photograph. Abnormal aneurysms and telangiectatic vessels in the temporal peripheral retina. Hard exudates and exudative changes in the posterior pole. **b)** FA. Three minutes after intravenous injection of fluorescein. **c)** FA. Twelve minutes after intravenous injection of fluorescein. Leakage of dye from abnormal vessels were observed, which increased in the late phase. **d)** Optical coherence tomography. Symbols indicate subfoveal serous retinal detachment site. Foveal macular thickness is 430 μm.

A 15-year-old boy had decreased visual acuity (VA) in his right eye since October 1, 2010. At his initial visit to our hospital, his VA was 0.4 OD. Aneurysms and abnormal telangiectatic vessels were observed at the temporal retina. Hard exudates and an exudative retinal detachment extended to the fovea (Figure 1a). Fluorescein angiography (FA) revealed hyperfluorescence corresponding to the abnormal telangiectatic lesion (Figure 1b) and increased leakage was seen at the late phase (Figure 1c). Optical coherence tomography (OCT; Cirrus™ OCT, Carl Zeiss, Meditec Inc., Dublin, CA, USA) indicated hard exudates in the macular area and subfoveal serous retinal detachment (Figure 1d). A 30 Hz flicker electroretinogram was non-recordable. He was diagnosed with Coats’ disease at stage 3A and aneurysms and abnormal telangiectatic retinal vessels were treated immediately with retinal photocoagulation. The serous retinal detachment resolved and his VA improved to 1.0.

However, aneurysms and exudative changes were aggravated after 5 months. The serous detachment recurred and VA decreased to 0.1. Laser photocoagulation was added to the abnormal vessels; however, his VA declined to 0.06 on May 20, 2011. We concluded that laser retinal photocoagulation alone was not enough to gain sufficient effects. Therefore, on May 27, 2011, we added IVB (Avastin®; 1.25 mg/0.05 mL) injection into the vitreous through the pars plana under local anesthesia. Exudative change in the retina was found to be eased at a clinic visit on June 10, 2011, which was 14 days after IVB injection. Therefore, laser photocoagulation therapy was given to cover the abnormal capillaries, aneurysms and the leakage area spotted in FA.

**Figure 2:**
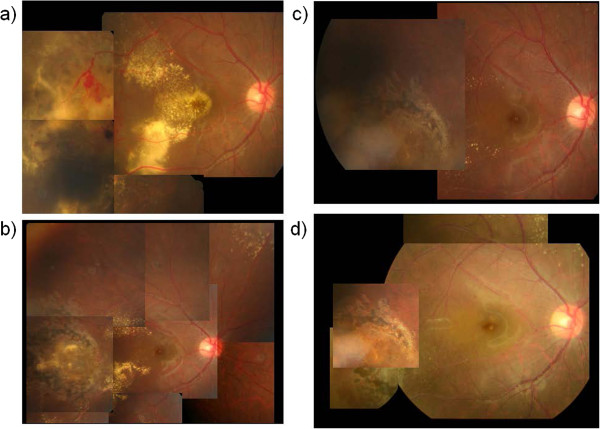
**Case 1 fundus photographs. a)** Prior to the injection of intravitreal bevacizumab (IVB). **b)** Five months after IVB injection. Hard exudates in the posterior pole had gradually resolved. **c)** Eleven months after IVB injection. Hard exudates in the posterior pole had almost disappeared. **d)** Eighteen months after IVB injection. Abnormal retinal vessels in the periphery are completely cicatrized and no recurrence is observed.

Three months after IVB, best corrected visual acuity (BCVA) improved to 0.3, and the lesions cicatrized. Six months after IVB, BCVA improved to 0.6 and OCT revealed that the serous retinal detachment had resolved. The hard exudates and macular edema decreased remarkably. There was no recurrence for more than 2 years and BCVA gradually improved to 1.2 in 1 year. Fundus findings are shown in Figure 2.

**Figure 3:**
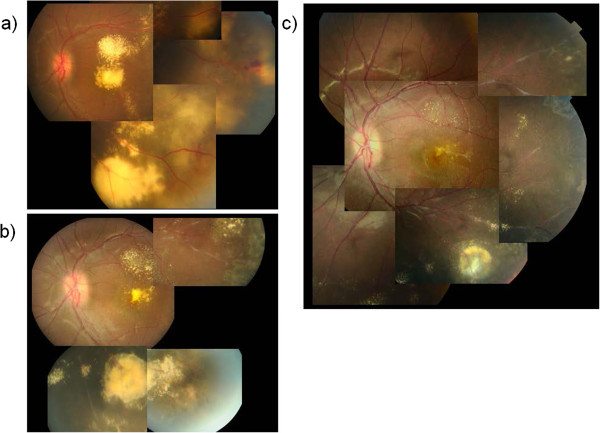
**Case 2 fundus photographs. a)** Prior to IVB injection. Abnormal telangiectatic vessels in the temporal retina and accumulation of massive subfoveal hard exudates. **b)** Six months after IVB injection. Hard exudates and retinal edema in the posterior pole were decreased. **c)** Fourteen months after IVB injection. Subfoveal hard exudates had almost resolved, retinal edema was alleviated, and temporal telangiectasis was cicatrized.

**Figure 4:**
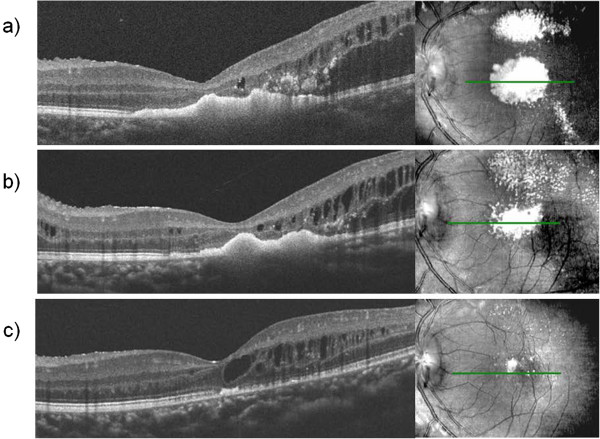
**Case 2 OCT. a)** Prior to IVB injection. Massive accumulation of subfoveal hard exudates, cystoids macular edema and partial serous retinal detachment were observed. **b)** Two months after IVB injection. Subfoveal hard exudates were slightly reduced. **c)** Eleven months after IVB injection. Subfoveal hard exudates had almost resolved. The ELM line disruption was repaired.

Case 2 was an 11-year-old boy with decreased VA of 0.15 OS at the initial visit on May 22, 2012. Abnormal telangiectatic vessels stretched widely in the temporal retina and massive hard exudates and retinal edema were found in the posterior pole. Hard exudates also accumulated in the fovea of his left eye. FA revealed telangiectatic capillaries in the temporal retina. OCT findings included massive hard exudates, macular edema and serous retinal detachment. The subfoveal external limiting membrane (ELM) and inner segment/outer segment (IS/OS) were disrupted. Our diagnosis was stage 3A Coats’ disease. Extensive photocoagulation was performed as the initial treatment to the lesion followed by phased laser therapy seven times to the lesion in the temporal retina. However, the exudative changes were severe and resistant to laser photocoagulation therapy (Figures 3a, 4a). Therefore, we decided to add bevacizumab as an adjuvant before the photocoagulation. On September 7, 2012, we added an IVB (Avastin®; 1.25 mg/0.05 mL) injection into the vitreous through the pars plana under local anesthesia. The exudative changes seemed to improve 7 days after IVB injection on September 15, 2012 and therefore phased laser phototherapy was added to cover the abnormal capillaries every 1 month, with a total of three treatments. After combination therapy, retinal exudation was remarkably decreased and hard exudates gradually resolved. Three months after IVB, VA improved to 0.5, and 6 months after IVB, submacular hard exudates showed marked reduction. BCVA improved to 0.7. One year after IVB, the hard exudates gradually resolved and no recurrence was observed. Fundus photos and OCT findings are shown in Figures 3 and 4, respectively. Along with the regression of retinal exudative changes, subfoveal hard exudates were reduced and a subfoveal absence of an ELM and IS/OS line were repaired. The BCVA gradually improved.

These treatment protocols were approved by the IRB/Ethics Committee at Kinki University and followed the tenets of the Declaration of Helsinki. Both patients and their parents gave written informed consents after explanation of the nature and possible consequences of the procedure.

## Discussion

VEGF is well known to be involved in the etiology of ocular disorders like exudative age-related macular degeneration (AMD), diabetic retinopathy, retinal vein occlusion (RVO), myopic choroidal neovascularization and neovascular glaucoma. Intravitreal injection of anti-VEGF is widely used for treatment of the above-mentioned diseases. Moreover, the range of disease that could benefit from anti-VEGF therapy has widened.

Coats’ disease most often affects young boys, who are often too young to report their symptoms. Therefore, when the patient visits an eye clinic after noticing clear ocular disorder, the condition is already at advanced stages with severe exudative changes. Morris et al. [12] reported that most cases of the 55 eyes investigated were in stage 2 or stage 3 according to Shields classification (20% were in stage 2A; about 42% were in stage 2B; about 27% were in stage 3A; about 8% were in stage 3B; there were no eyes in stage 1 or stage 5 and only four eyes in stage 4).

There are several treatment modalities for Coats’ disease, depending on its stage and the patient’s age. A first choice for treating Coats’ disease is photocoagulation or cryotherapy, and it is considered that independent application of either modality is effective at stage 1. However, it is very rare that Coats’ disease is diagnosed at stage 1, as described in previous reports. The most frequent cases of stage 2 to stage 3A do not respond well to their independent application because retinal exudative change and serous retinal detachment are present at advanced stages of the disease, which obstruct laser therapy. To drain subretinal fluid before photocoagulation, surgical intervention might be necessary, or, alternatively, anti-VEGF injection as an adjuvant therapy might be effective. Case 1 had severe retinal exudative changes and was resistant to laser photocoagulation therapy, although there was a favorable response to bevacizumab injection with a good prognosis. Case 2 had extended retinal exudation with massive subfoveal hard exudates. After the injection of bevacizumab was added as a treatment, the focus was completely cicatrized and hard exudates gradually resolved. In this study, we demonstrated that this combined therapy could be a very effective treatment for the most frequent cases of Coats’ disease in stage 2 to 3A. Ramasubramanian et al. [13] reported IVB injection caused vitreoretinal fibrosis in four of eight patients, with three evolving to traction retinal detachment in the short-term follow-up of 8.5 months. We therefore needed to judge the indications very carefully. Though we did not observe complications in our studies, it would be prudent to pay attention to additional complications such as vitreoretinal fibrosis that progress to retinal detachments following IVB treatments.

Morris et al. [12] reported that stage 3 or worse is most common diagnosis for very young children and less common in older patients. Individual consideration should be given to patients with regard to anesthesia and treatment methods according to age. To effectively apply laser therapy, patient compliance during treatment is indispensable. Therefore, very young cases which need general anesthesia, might not be suitable if they need frequent laser therapy. Our two cases were 15 years old and 11 years old and were therefore old enough to undergo IVB injection and laser therapy under local anesthesia. They were also cooperative during diagnosis and treatment. Their willingness to cooperate was one of the factors that allowed a good prognosis.

Ito et al. [14] reported that, in DMO, the ELM status may be as closely related to VA as the IS/OS status. In Case 2, OCT was used to observe changes in the retinal outer layer to identify the repaired ELM and IS/OS, improved vision and resolved hard exudates. The cases in the present study had long-term hard exudates in the macular area; however, they probably did not reach an irreversible level of structural damage. Future investigation is necessary to determine the duration that causes an irreversible level of damage on the ocular structures leading to irreversible visual loss.

## Conclusion

In conclusion, IVB injection can be an effective method of treatment for Coats’ disease that is resistant to photocoagulation therapy alone.

## Consent

Written informed consent was obtained from the patient for publication of this case report and any accompanying images.

## Abbreviations

IVB: Intravitreal bevacizumab; SRF: Subretinal fluid; VEGF: Vascular endothelial growth factor; VA: Visual acuity; BCVA: Best corrected visual acuity; FA: Fluorescein angiography; OCT: Optical coherence tomography; ELM: External limiting membrane; IS/OS: Inner segment/outer segment.

## Competing interests

The authors declare that they have no competing interests.

## Authors’ contributions

AK carried out substantial part of the work by diagnosing the patients, treating and following up them, analyzing the acquired data and made a draft manuscript to finalize it upon suggestions and advice from the other authors. KS participated in the treatment by carrying out IVB injection and laser photocoagulation in the patients and contributed in revising the manuscript critically for important intellectual content. SK and CM participated in the treatment by giving advice and recommendations. YS helped to finalize the manuscript and gave final approval of the version to be published. All authors read and approved the final manuscript.

## Pre-publication history

The pre-publication history for this paper can be accessed here:

http://www.biomedcentral.com/1471-2415/14/36/prepub
